# Prevalence of congenital malaria in high-risk Ghanaian newborns: a cross-sectional study

**DOI:** 10.1186/1475-2875-12-17

**Published:** 2013-01-11

**Authors:** Christabel C Enweronu-Laryea, George O Adjei, Benjamin Mensah, Nancy Duah, Neils B Quashie

**Affiliations:** 1 Department of Child Health, University of Ghana Medical School, P O Box 4236, Accra, Ghana; 2 Centre for Tropical Clinical Pharmacology and Therapeutics, University of Ghana Medical School, Accra, Ghana; 3 Department of Haematology, Korle Bu Teaching Hospital, Accra, Ghana; 4 Department of Epidemiology, Noguchi Memorial Institute for Medical Research, University of Ghana, Accra, Ghana

**Keywords:** Congenital, Malaria, Newborn, Ghana

## Abstract

**Background:**

Congenital malaria is defined as malaria parasitaemia in the first week of life. The reported prevalence of congenital malaria in sub-Saharan Africa is variable (0 - 46%). Even though the clinical significance of congenital malaria parasitaemia is uncertain, anti-malarial drugs are empirically prescribed for sick newborns by frontline health care workers. Data on prevalence of congenital malaria in high-risk newborns will inform appropriate drug use and timely referral of sick newborns.

**Methods:**

Blood samples of untreated newborns less than 1 week of age at the time of referral to Korle Bu Teaching hospital in Accra, Ghana during the peak malaria seasons (April to July) of 2008 and 2010 were examined for malaria parasites by, i) Giemsa-stained thick and thin blood smears for parasite count and species identification, ii) histidine-rich protein- and lactic dehydrogenase-based rapid diagnosis tests, or iii) polymerase chain reaction amplification of the merozoite surface protein 2 gene, for identification of sub-microscopic parasitaemia. Other investigations were also done as clinically indicated.

**Results:**

In 2008, nine cases of *Plasmodium falciparum* parasitaemia were diagnosed by microscopy in 405 (2.2%) newborns. All the nine newborns had low parasite densities (≤50 per microlitre). In 2010, there was no case of parasitaemia by either microscopy or rapid diagnosis tests in 522 newborns; however, 56/467 (12%) cases of *P*. *falciparum* were detected by polymerase chain reaction.

**Conclusion:**

Congenital malaria is an uncommon cause of clinical illness in high-risk untreated newborns referred to a tertiary hospital in the first week of life. Empirical anti-malarial drug treatment for sick newborns without laboratory confirmation of parasitaemia is imprudent. Early referral of sick newborns to hospitals with resources and skills for appropriate care is recommended.

## Background

Malaria in pregnancy is a major underlying cause of neonatal morbidity in endemic regions [1-3]. The burden of malaria in pregnancy is highest in sub-Saharan Africa where 1:4 pregnant women have evidence of placental infection at the time of delivery [4-6]. Pregnancy associated malaria has been implicated as a cause of stillbirths, premature births, intrauterine growth restriction and consequently low birth weight (LBW) in affected newborns. However, its effect as a cause of clinical disease in the newborn especially in the first week of life, when most newborn illnesses and deaths occur, is uncertain.

Congenital malaria, defined as malaria parasitaemia in the first week of life can be acquired transplacentally or during the time of birth. The prevalence in sub-Saharan Africa has been reported to vary from 0 – 46% [7]; however, more recent multicentre longitudinal studies have reported lower rates of 4 – 6% [8,9]. Clinical malaria is rare in newborns from endemic areas with high incidence of malaria in pregnancy. This rare occurrence has been attributed to the transplacental transfer of maternally derived antibodies to malaria, and the inhibitory effect of foetal haemoglobin on malaria parasite development [10-12].

The prevalence of congenital malaria in a community based prospective study in infants in southern Ghana in 1998 was 13.6% by polymerase chain reaction (PCR) and 2.5% by microscopy [13]. In that study the risk of infection was three times higher during the rainy season (April – July) as compared to the dry season. Recently, two cases of symptomatic congenital malaria were reported in Ghanaian newborns [14,15].

Clinical malaria in the newborn usually manifests after the first week of life (but has been reported on day 1) with symptoms such as fever, poor feeding, lethargy, anaemia, hepatosplenomegaly, jaundice, irritability, and drowsiness. These clinical features are similar to the manifestations of neonatal sepsis and other neonatal conditions that more commonly cause morbidity and mortality in the newborn period [8,16,17]. Many malaria endemic sub-Saharan African countries lack quality laboratory equipment and skilled frontline health care workers in primary health care facilities. As such, diagnosing neonatal problems is a major challenge and empirical treatment for malaria is often prescribed when newborns first present with the above symptoms. Sick newborns are referred to hospital few days later when malaria treatment does not resolve symptoms. This practice results in delayed referral of sick newborns for appropriate care and contributes to the high neonatal mortality and long-term morbidity in survivors in the sub-region.

Malaria is endemic in Ghana and World Health Organization (WHO) guidelines for empirical treatment of malaria in suspected cases is widely adhered to [18]. This practice is extended to sick newborns by primary care health workers contrary to existing guidelines on the Integrated Management of Childhood Illnesses. Though many underlying reasons may account for this practice in sick newborns, the lack of sufficient local data on congenital malaria may be a contributory factor. This study presents the prevalence of malaria parasitaemia in non-treated high-risk newborns referred to a teaching hospital in the first week of life.

## Methods

This cross-sectional study was done at the newborn unit and malaria research laboratory of the Department of Child Health, Korle Bu Teaching Hospital, in Accra during the peak malaria seasons (April – July) of 2008 and 2010. The 55-bed newborn unit serves as a tertiary referral centre for sick and low birth weight or preterm newborns from southern regions of Ghana. About 2000 newborns are admitted annually. Ethical approval was given by the Ethical and Protocol Review Committee of the University of Ghana Medical School.

All newborns hospitalized at the newborn unit in the first week of life during the study period were eligible for inclusion. Newborns with clinical indications that required blood sampling e.g. signs of illness or risk factors for infection were enrolled. Stable newborns e.g. infants of diabetic mothers or well preterm infants of hypertensive mothers who were referred for observation and did not require laboratory investigations, were excluded. All clinical procedures and laboratory investigations for hospitalized newborns were done according to standard and departmental guidelines.

A structured questionnaire was used to collect clinical data on newborn and maternal health during pregnancy, including use of insecticide treated nets and intermittent preventive treatment. Other clinical data was extracted from the ward register and hospital files. Sample size calculation was based on an estimated prevalence of a 20% risk of congenital malaria in high-risk newborns in the first week of life [2,11].

### Laboratory investigations

Newborn venous blood (1 ml) was collected into EDTA tubes for haematological investigations. Total white blood cell count (WBC) with differential, platelet count and red cell distribution width was measured by an automated haematology analyzer (Sysmex KX-21) according to manufacturer’s instructions. Thick and thin blood smears prepared from the EDTA sample were stained with Giemsa. Thin smears were used for malaria parasite species identification, and the number of asexual parasites per 200 white blood cells (WBC) on the thick smear was multiplied by the measured WBC count to obtain the parasite count per microlitre. An experienced technologist conducted periodic controls of blood slides for quality control. This traditional method of microscopy was used in 2008 and 2010. In 2010, a rapid diagnosis test (RDT) for identification of malaria parasite histidine rich protein 2 and lactate dehydrogenase (the First Response® malaria antigen pLDH/HRP2 Combo Test), and a nested PCR methods were used to identify sub-microscopic parasitaemia.

### RDT

The First Response® malaria antigen pLDH/HRP2 Combo Test (Premier Medical Corporation Ltd, India) is designed for the differential diagnosis between *Plasmodium falciparum* and other plasmodial species. It contains a membrane strip pre-coated with two monoclonal antibodies as two separate lines across a test strip. One monoclonal antibody is pan-specific to lactate dehydrogenase of the Plasmodium species and the other monoclonal antibody is specific to histidine-rich protein 2 of *P. falciparum*[19].

### PCR

This was done at the Noguchi Memorial Institute for Medical Research, University of Ghana. Parasite DNA was extracted from whole blood samples spotted on filter paper, using the TE method [20]. A nested PCR method for amplification of the merozoite protein 2 (MSP) gene was used for the detection of *P. falciparum*. The primary amplification reaction involved the use of the primer pair, S2 (5’-5’ GAG GGA TGT TGC TGC TCC ACA G 3’) for a 450-800 base pair product. The PCR product obtained was used as the template for the secondary PCR using the following primer pairs: S1 (5’-GAG TAT AAG GAG AAG TAT G-3’) and S4 (5’-CTA GAA CCA TGC ATA TGT CC-3’) for a 400-700 base pair product. The PCR reactions were carried out in a final volume of 25 μl, which contained 1xPCR buffer (50 mM KCl, 20 mM Tris-HCl, pH 8.3, 0.1 mg/ml of gelatin), 3.5 mM MgCl_2_, 200 μM dNTP mix, 1 μM of each primer, 1U Taq polymerase and 5 μl of DNA template for primary amplification. The secondary reaction contained 1xPCR buffer (50 mM KCl, 20 mM Tris-HCl, pH 8.3, 0.1 mg/ml of gelatin), 2.5 mM MgCl_2_, 200 μM dNTP mix, 1 μM of each primer, 1U Taq polymerase and 1μl of DNA amplicon. PCR was carried out using a DNA Engine Tetrad PTC-225 thermal cycler (MJ Research, USA) with cycling parameters of an initial melt at 94°C for 3mins followed by 30 cycles of 94°C for 25secs, 42°C for 1 min, 65°C for 2 minutes and a final cycle of 72°C for 3mins. The cycling parameters for the secondary reaction was 94°C for 3mins followed by 30 cycles of 94°C for 25secs, 50°C for 1 min, 70°C for 2mins and a final cycle of 72°C for 3mins. PCR products were run on a 2% ethidium bromide stained agarose gel. The PCR assay detects one parasite per microlitre of blood and has about 97% sensitivity and 100% specificity to detect *P. falciparum.*

### Data analysis

Laboratory data was entered into a designated register. All data were subsequently entered into a database (Access 2007 for Windows, Microsoft Corporation, USA). Data analysis was by descriptive and inferential statistics. Pearson's chi square test (or Fisher's exact test as appropriate) was used to compare proportions.

## Results

In 2008, 405 newborns aged 0 – 7 days were tested in the first week of life. In 2010, 522 newborns admitted to the unit in the first week of life were tested during the course of their hospitalization (aged 0 – 28 days), of these 443 (84.9%), were tested in the first week of life. The number and selected demographic and clinical characteristics of newborns admitted during both study periods are shown in Table 1. There was no difference in the morbidity pattern of newborns during both periods.

**Table 1:** Characteristics of newborns tested for congenital malaria in 2008 and 2010

	**Newborns in 2008**	**Newborns in 2010**
	**n = 405 (%)**	**n = 522 (%)**
**Birth weight**		
< 2500 grams (LBW)	223 (55)	267 (51)
≥ 2500 grams	182 (45)	255 (49)
**Reason for admission**		
Prematurity/LBW	161 (39.8)	185 (35.4)
Suspected infection	119 (29.4)	152 (29.1)
Perinatal asphyxia	78 (19.3)	110 (21.1)
Jaundice	31 (7.7)	43 (8.2)
Other (birth injury, congenital anomalies, intrapartum maternal death)	15 (3.7)	32 (6.1)
**Laboratory data**		
Mean haemoglobin (g/dl)	15.5 (range 4.1 – 23.7)	15.4 (range 5.7 – 23)
Mean white blood cell count (x 10^9^)	10.4 (range 2.1 – 37.4)	12.8 (range 3 – 23)

There was no difference between subjects’ characteristics in 2008 as compared to 2010 (*p* > 0.05 for all parameters).

Nine (2.2%) of the 405 newborns tested in 2008 had *P. falciparum* parasitaemia by microscopy, all of which were of low parasite density (≤50 per μl). Four (44%) of the nine newborns were LBW and also born prematurely. Of the nine affected newborns, two were asymptomatic preterm LBW newborns who needed special care, three had suspected sepsis (they had no fever) because of risk factors for neonatal infection in their mothers’ obstetric history, three had perinatal asphyxia and one newborn was admitted on the 7^th^ day of life with low grade fever and moderate jaundice. The mean haemoglobin and WBC in newborns with congenital malaria was [15.5 g/dl (range 13.2 – 17.8)] and [10.5 × 10^9^ (range 4.2 – 19.4)] respectively. There was no statistical difference in the haematological indices of congenital malaria cases as compared with non-affected newborns (see Table 1). Affected newborns were treated with amodiaquine 10 mg/kg/day according to departmental guidelines.

None of the 522 newborns recruited in 2010 had parasitaemia by microscopy or RDT. All 522 samples were tested by PCR, results were obtained in 467/522 and 56 (12%) newborns were positive for *P. falciparum*. The prevalence of LBW in the PCR parasitaemia-positive newborns was 55% as compared to 41.9% in those who were PCR parasitaemia-negative [p = 0.1; RR 1.3 (95% CI 0.9 – 1.74)]. The mean haemoglobin (14.6 g/dl vs. 15.3 g/dl) and WBC (14.6 × 10^9^ vs. 16.4 × 10^9^) in those with parasitaemia vs. those without parasitaemia respectively was not statistically different.

Data collected from mothers about their health and malaria preventive practices (use of insecticide treated nets and intermittent preventive treatment) during pregnancy were not analyzed because the information could not be verified.

## Discussion

This study shows that patent malaria parasitaemia was uncommon in untreated high-risk newborns referred to Korle Bu Teaching hospital in southern Ghana during the peak seasons for malaria in 2008, and 2010. The 2.2% congenital malaria prevalence by microscopy in the 2008 cohort was lower than rates from longitudinal studies from neighbouring countries, 5.1% in Nigeria and 4.7% in Ivory Coast [8,9]. The data also suggests that congenital malaria is mostly asymptomatic with no pathognomic clinical features and that affected newborns have low parasitaemia; these findings are similar to work reported by others [21,22].

Several hospital-based studies from the West African sub-region have reported high prevalence of malaria in newborns [23,24]. The reasons for the variable prevalence of congenital malaria in these countries of similar endemicity as Ghana are unclear. Possible explanations could be differences in malaria prevention coverage during pregnancy or laboratory operational factors. Traditional microscopy in routine service laboratories may over-diagnose malaria, and has been shown to have a poor positive predictive value when compared with microscopy from research laboratories [25,26].

The PCR method diagnoses congenital malaria more frequently because of its ability to detect parasite macromolecules and not necessarily live parasites [21]. The clinical importance and significance of malaria parasitaemia diagnosed by PCR alone is uncertain [27]. Even though it is suggestive of placental parasitaemia and the consequent effects of LBW and prematurity but its relevance as a significant cause of clinical illness in the first week of life is doubtful. However, recent evidence suggests that *P. falciparum* malaria parasites in the placenta as detected by PCR are more likely to result in congenital malaria in the infant during the first three months of life [28].

Most widely used RDTs are based on the detection of the histidine-rich protein 2 (HRP2) product of the *P. falciparum hrp2* gene. The sensitivity and specificity of this method has been shown to range from 90-95% and 85-92% respectively. However, relative to thick smear microscopy, recent studies have shown that false negative RDT results occur in persons with asymptomatic *P. falciparum* infections as in our patients. The false negatives are thought to result from spontaneous deletion of the histidine-rich repeat region of the *hrp2* gene [29].

The data from this study and previous community-based study on congenital malaria in Ghana [15] shows that congenital malaria is an uncommon cause of neonatal morbidity in Ghana. A large-scale hospital-based surveillance study in Kenya from 2002 - 2009 found a declining trend in the prevalence of congenital malaria; newborns (0.5%) who had malaria parasitaemia presented before 2005 [30]. Other workers have also shown a declining trend in malaria disease in recent years [31,32]. These studies suggest that clinical congenital malaria is not a significant cause of the high neonatal morbidity and mortality in sub-Saharan Africa. It is therefore important that sub-Saharan African countries with endemic malaria should urgently review newborn care guidelines for primary health care workers so as to reduce delays in the referral and appropriate management of sick newborns.

This study has some limitations. Though one of the objectives was to identify the impact of maternal malarial preventive practices in pregnancy on the prevalence of congenital malaria, we could not collect accurate data on maternal practices. It was difficult to verify that those who said they had used insecticide treated nets during pregnancy actually did so. Also, the information mothers gave on intermittent anti-malarial prophylaxis was not always recorded in the available maternal health records.

## Conclusion

The data from this study does not support empirical treatment for malaria in sick newborns in Ghana. Laboratory confirmation of malaria in newborns should be done and other causes of clinical illness excluded before anti-malarial drugs are prescribed for newborns especially in the first week of life. The prevalence of malaria as detected by PCR in this study could be used to make a case for improving malaria preventive practices during pregnancy thus reducing placental malaria - a known cause of LBW and a pre-requisite for congenital malaria. Early referral of sick newborns to health facilities with adequate skills for early diagnosis and appropriate management is recommended. Concerted effort to reduce empirical prescription of anti-malarial drugs for newborns is needed. Data such as presented in this study could be used in continuing educational programs to improve knowledge and influence the practice of frontline health care providers.

## Abbreviations

LBW: Low birth weight; PCR: Polymerase chain reaction; RDT: Rapid diagnostic test; WBC: White blood cells; WHO: World Health Organization.

## Competing interests

The authors have no competing interest to declare. None of the authors received any remuneration for this work. The laboratory investigations of this study were partly supported by DANIDA Denmark (DFC grant number 09-080RH).

## Authors’ contribution

CEL conceived the study and worked with GOA and BM on the design and data acquisition. GOA, BM, ND and NBQ did the laboratory work. CEL drafted the manuscript and all 5 authors participated in the several revisions of the manuscript and gave approval for the final version for publication.

## References

[B1] Brabin BJ An analysis of malaria in pregnancy in Africa Bull World Health Organ 1983 61 1005 1016 6370484 PMC2536236

[B2] Fischer PR Congenital malaria: an African survey Clin Pediatr 1997 36 411 413 10.1177/000992289703600706 9241479

[B3] Breman JG Alilio MS Mills A Conquering the intolerable burden of malaria: what's new, what's needed: a summary Am J Trop Med Hyg 2004 7 Suppl 2 1 15 15331814

[B4] Desai M ter Kuile FO Nosten F McGready R Asamoa K Brabin B Newman RD Epidemiology and burden of malaria in pregnancy Lancet Infect Dis 2007 7 93 104 17251080 10.1016/S1473-3099(07)70021-X

[B5] Guyatt HL Snow RW Impact of malaria during pregnancy on low birth weight in sub-Saharan Africa Clin Microbiol Rev 2004 17 760 769 15489346 10.1128/CMR.17.4.760-769.2004 PMC523568

[B6] Guyatt HL Snow RW Malaria in pregnancy as an indirect cause of infant mortality in sub-Saharan Africa Trans R Soc Trop Med Hyg 2001 95 569 76 11816423 10.1016/s0035-9203(01)90082-3

[B7] Uneke CJ Congenital *Plasmodium falciparum* malaria in sub-Saharan Africa: a rarity or frequent occurrence J Parasitol Res 2007 101 835 842 10.1007/s00436-007-0577-9 17549517

[B8] Orogade AA Falade CO Okafor HU Mokuolu OA Mamman AI Ogbonu TA Ogunkunle OO Ernest KS Callahan MV Hamer DH Clinical and laboratory features of congenital malaria in Nigeria Pediatr Infect Dis J 2008 3 181 187

[B9] Vanga-Bosson HA Coffie PA Kanhon S Sloan C Kouakou F Eholie SP Kone M Dabis F Menan H Ekouevi DK Coverage of intermittent prevention treatment with sulphadoxine-pyrimethamine among pregnant women and congenital malaria in Côte d'Ivoire Malar J 2011 10 105 21529344 10.1186/1475-2875-10-105 PMC3108318

[B10] Gitau GM Eldred JM Malaria in pregnancy: clinical, therapeutic and prophylactic considerations Obstet Gynecol 2005 7 5 11

[B11] WHO Severe falciparum malaria Trans R Soc Trop Med Hyg 2000 94 Suppl 1 1 90 11103309

[B12] Snow RW Nahelen B Palmer A DonNelly CA Gupta S Marsh K Risk of severe malaria among African infants: direct evidence of clinical protection during early infancy J Infect Dis 1998 3 819 822 9498474 10.1086/517818

[B13] Wagner G Koram K Mcguinness D Bennett S Nkrumah F Riley E High incidence of asymptomatic malaria infections in a birth cohort of children less than one year of age in Ghana, detected by multicopy gene polymerase chain reaction Am J Trop Med Hyg 1998 59 115 123 9684638 10.4269/ajtmh.1998.59.115

[B14] Opare DA Congenital malaria in newborn twins Ghana Med J 2010 44 76 78 21327008 10.4314/gmj.v44i2.68888 PMC2994153

[B15] Zenz W Trop M Kollaritsch H Reinthaler F Congenital malaria due to *Plasmodium falciparum* and *Plasmodium malariae* Wien Klin Wochenschr 2000 112 459 461 10890139

[B16] Opiyo N English M What clinical signs best identify severe illness in young infants aged 0–59 days in developing countries? a systematic review Arch Dis Child 2011 96 1052 1059 21220263 10.1136/adc.2010.186049 PMC3081806

[B17] Mazzi E Bartos AE Carlin J Weber MW Darmstadt GL Bolivia Clinical Signs Study Group: Clinical signs predicting severe illness in young infants (<60 days) in Bolivia J Trop Pediatr 2010 56 307 316 20144933 10.1093/tropej/fmq005

[B18] World Health Organization Implementation of the Global Malaria Strategy WHO Geneva Technical record Series no. 83 1993 Geneva: WHO

[B19] Iqbal J Hira PR Sher A Al-Enezi AA Diagnosis of imported malaria by Plasmodium Lactate Dehydrogenase (pLDH) and histidine-rich protein 2 (HRP2)-based immunocapture assays Am J Trop Med Hyg 2001 64 20 23 11425156 10.4269/ajtmh.2001.64.20

[B20] Bereczky S Mårtensson A Pedro Gil J Farnert A Rapid DNA extraction from archive blood spots on filter paper for genotyping of *Plasmodium falciparum* Am J Trop Med Hyg 2005 72 249 251 15772315

[B21] Kassberger F Birkenmaier A Khattab A Kremsner PG Klinkert MQ PCR typing of *Plasmodium falciparum* in matched peripheral, placental, and umbilical cord blood J Parasitol Res 2002 88 1073 1079 10.1007/s00436-002-0715-3 12444458

[B22] Brabin BJ Congenital Malaria – a recurrent problem Ann Trop Paediatr 2007 27 95 98 17565805 10.1179/146532807X192453

[B23] Mukhtar MY Lesi FE Iroha EU Egri-Okwaji MT Mafe AG Congenital malaria among inborn babies at a tertiary centre in Lagos Nigeria J Trop Pediatr 2006 52 19 23 10.1093/tropej/fmi044 15927946

[B24] Uneke CJ Congenital malaria: an overview Tanzan J Health Res 2011 13 264 280

[B25] Reyburn H Mbatia R Drakeley C Carneiro I Mwakasungula E Mwerinde O Saganda K Shao J Kitua A Olomi R Greenwood BM Whitty CJ Overdiagnosis of malaria in patients with severe febrile illness in Tanzania: a prospective study BMJ 2004 329 1212 15542534 10.1136/bmj.38251.658229.55 PMC529364

[B26] Oduwole OA Oduwole OA Ejezie GC Odey FA Oringanje CM Nwakanma D Bello S Oriero E Okebe J Alaribe AA Etuk S Meremikwu M Congenital malaria in Calabar, Nigeria: the molecular perspective Am J Trop Med Hyg 2011 84 386 389 21363974 10.4269/ajtmh.2011.10-0253 PMC3042812

[B27] Coll O Menendez F Botet Dayal R World Association of Perinatal Medicine Perinatal Infections Working Group Treatment and prevention of malaria in pregnancy and newborn J Perinat Med 2008 36 15 29 18184095 10.1515/JPM.2008.002

[B28] Mwangoka GW Kimera SI Mboera LEG Congenital *Plasmodium falciparum* infection in neonates in Muheza district Tanzania Malar 2008 7 117 10.1186/1475-2875-7-117 PMC2474641 18598342

[B29] Koita OA Doumbo OK Ouattara A Tall LK Konaré A Diakité M Diallo M Sagara I Masinde GL Doumbo SN Dolo A Tounkara A Traoré I Krogstad DJ False-negative rapid diagnostic tests for malaria and deletion of the histidine-rich repeat region of the hrp2 Gene Am J Trop Med Hyg 2012 86 194 198 22302847 10.4269/ajtmh.2012.10-0665 PMC3269266

[B30] Mwaniki MK Talbert AW Mturi FN Berkley JA Kager P Marsh K Newton CR Congenital and neonatal malaria in a rural Kenyan district hospital. an eight-year analysis Malar J 2010 9 313 21054891 10.1186/1475-2875-9-313 PMC2988044

[B31] Okiro EA Alegana VA Noor AM Mutheu JJ Juma E Snow RW Malaria paediatric hospitalization between 1999 and 2008 across Kenya BMC Med 2009 7 75 20003178 10.1186/1741-7015-7-75 PMC2802588

[B32] Sievers AC Lewey J Musafiri P Franke MF Bucyibaruta BJ Stulac SN Rich ML Karema C Daily JP Reduced paediatric hospitalizations for malaria and febrile illness patterns following implementation of community-based malaria control programme in rural Rwanda Malar J 2008 7 167 18752677 10.1186/1475-2875-7-167 PMC2557016

